# Enhanced cervical precancerous lesions detection and classification using Archimedes Optimization Algorithm with transfer learning

**DOI:** 10.1038/s41598-024-62773-x

**Published:** 2024-05-27

**Authors:** Ayed S. Allogmani, Roushdy M. Mohamed, Nasser M. Al-shibly, Mahmoud Ragab

**Affiliations:** 1 University of Jeddah, College of Science and Arts at Khulis, Department of Biology, Jeddah, Saudi Arabia; 2 Physiotherapy Department, College of Applied Health Sciences, Jerash University, Jerash, Jordan; 3 Information Technology Department, Faculty of Computing and Information Technology, King Abdulaziz University, 21589 Jeddah, Saudi Arabia

**Keywords:** Cervical cancer, Human papillomavirus, Archimedes Optimization Algorithm, Transfer learning, Medical image, Computer science, Information technology

## Abstract

Cervical cancer (CC) ranks as the fourth most common form of cancer affecting women, manifesting in the cervix. CC is caused by the Human papillomavirus (HPV) infection and is eradicated by vaccinating women from an early age. However, limited medical facilities present a significant challenge in mid- or low-income countries. It can improve the survivability rate and be successfully treated if the CC is detected at earlier stages. Current technological improvements allow for cost-effective, more sensitive, and rapid screening and treatment measures for CC. DL techniques are widely adopted for the automated detection of CC. DL techniques and architectures are used to detect CC and provide higher detection performance. This study offers the design of Enhanced Cervical Precancerous Lesions Detection and Classification using the Archimedes Optimization Algorithm with Transfer Learning (CPLDC-AOATL) algorithm. The CPLDC-AOATL algorithm aims to diagnose cervical cancer using medical images. At the preliminary stage, the CPLDC-AOATL technique involves a bilateral filtering (BF) technique to eliminate the noise in the input images. Besides, the CPLDC-AOATL technique applies the Inception-ResNetv2 model for the feature extraction process, and the use of AOA chose the hyperparameters. The CPLDC-AOATL technique involves a bidirectional long short-term memory (BiLSTM) model for the cancer detection process. The experimental outcome of the CPLDC-AOATL technique emphasized the superior accuracy outcome of 99.53% over other existing approaches under a benchmark dataset.

## Introduction

CC arises once a cell inside the cervix develops quickly, creating a malignant cell. An HPV virus is responsible for developing tumour cells^1^. A Pap smear test is more time-consuming because it needs a radiologist's analysis of a few 10,000 cells to recognize all cell abnormalities^2^. Accordingly, current healthcare will be changed to artificial intelligence (AI) and DL for identifying and diagnosing CC^3^. An automated method can also recognize cancer cells in a fraction of the time; however, it will provide objective and precise outcomes^4^. Once the tumour develops malignant, the cell spreads to various body parts; specific areas become resulting infections and can be prevented via earlier identification in primary severe conditions. Mortality owing to cervical malignancies will be decreased when efficient screening approaches have been executed^5^. With the rapid growth of current medical and computer technological innovations, numerous diagnostic and screening methods rely on computer-aided (CAD) systems. Machine learning (ML) enhances the performance of analyses and produces accurate patient data^6^. One research work utilized econometric tools, ML, and text mining to find which main and improved quality characteristics and reactions have been more related to predicting customer fulfilment in various service conditions^7^.

This article develops an automatic and ML-based method for producing insights. Additionally, the significance of constant quality enhancement in the effectiveness of ML techniques from a medical management and management information technologies viewpoint is proved in this study by reporting various categories of ML methods and analyzing clinical data through ML techniques^8,9^. This study recognized methods that are effectively suitable for classifying negative and positive CC for medical applications. CC must be diagnosed using these techniques. DL represents a major effect under health and medical imaging that supports an estimate of the diagnostic accuracy of DL techniques to recognize pathologies in medical imaging^10^. A DL model employing the Swin Transformer with an HSW-MSA module and a rescaled model for classification is presented. Also, ResMLP, replacing the conventional MLP, has been adopted^11^. A vision transformer method integrates squeeze-and-excitation and global response normalization-based MLPs^12^.

This study offers the design of Enhanced Cervical Precancerous Lesions Detection and Classification using Archimedes Optimization Algorithm with Transfer Learning (CPLDC-AOATL) methodology. The CPLDC-AOATL system aims to diagnose CC on the medical images. At the preliminary stage, the CPLDC-AOATL technique involves a bilateral filtering (BF) technique to get rid of the noise in the input images. Besides, the CPLDC-AOATL technique applies the Inception-ResNetv2 model for the feature extraction process, and the use of AOA chose the hyperparameters. The CPLDC-AOATL technique involves a bidirectional long short-term memory (BiLSTM) model for the cancer detection process. The experimental values of the CPLDC-AOATL method take place on a benchmark database.

- The CPLDC-AOATL model presents a bilateral filtering (BF) technique for efficiently removing noise from medical images, enhancing the quality of input data for subsequent evaluation.
- By implementing the Inception-ResNetv2 technique, the CPLDC-AOATL model implements a state-of-the-art deep learning model for feature extraction, enabling the capture of complex patterns and structures associated with detecting cervical cancer.
- The methodology's hyperparameters are carefully selected using the AOA technique, ensuring optimal accomplishment and generalization capability across diverse datasets and scenarios.
- By integrating a bidirectional long-short-term memory (BiLSTM) technique, the CPLDC-AOATL technique achieves superior accuracy in recognizing cervical cancer from medical images, outperforming existing techniques on benchmark datasets.

The remaining sections of the article are arranged as follows: "Literature review" section offers the literature review, and "The proposed method" section represents the proposed method. Then, "Performance validation" section elaborates on the results evaluation, and "Conclusion" section completes the work.

## Literature review

Nour et al.^13^ introduced a Computer Aided CC Diagnosis employing the Gazelle Optimizer Algorithm with DL (CACCD-GOADL) method. This technique deployed an enriched MobileNetv3 architecture for extraction. Moreover, the method develops an innovative GOA for the tuning process of the enhanced MobileNetv3 model. The method employs a stacked ELM (SELM) technique for classification. Tekchandani et al.^14^ developed the DL-based innovative and modified model dependent upon attention mechanism and residual theory with the base UNet architecture for the CLNs detection part (LNdtnNet) of the CADx model. Jeyshri and Kowsigan^15^ projected a method for segmenting multi-class cells into Nucleus and Cytoplasmic regions. A multi-resolution U-Net (MRU-Net) system was offered. Primarily, added semantic data was mined employing a series of recurrent convolutions. Secondarily, a convolutional module with different receptive domains was employed. The impact of network width unpredictability can be alleviated by incorporating a convolution layer with many corresponding domains. He et al.^16^ developed RMIL, an innovative registration-improved manifold instance learning pipeline. This can be needed only slide-level annotations and a smaller number of patch-level annotations for training. Moreover, self-supervised learning and attention mechanisms have been presented to improve feature extraction. In^17^, Raman spectroscopy was employed. CNN and ResNet classification methods could be made for classification. The attention mechanism squeeze-and-excitation network (SENet) and effective channel attention network (ECANet) units have been incorporated with the developed CNN and ResNet architectures. In^18^, DL methods are utilized. Then, an integrative method with DL techniques and ensemble methods like the maximal occurrence and possibility rate of cervical cells have been developed. The multi-cell analysis of the Pap smear image permitted combined forecasts of CC images employing the developed technique. The authors^19^ implemented an intelligent DCNN for CC detection and classification (IDCNN-CDC) system encompassing four primary techniques. The Gaussian filter (GF) and Tsallis entropy method with the dragonfly optimizer (TE-DFO) method are used for noise removel and segmentation.

The cell images have been provided to the DL-based SqueezeNet system for extraction. The authors^20^ projected a CAD for CC Screening employing Monarch Butterfly optimizer with DL (CADCCSMBODL) technique, which utilizes transfer learning with tuning approaches for classification. Adaptive filtering (AF) was also deployed with saliency-based segmentation techniques. Also, the method utilizes EfficientNet with the MBO method for parameter optimization. In conclusion, the XGBoost algorithm was implemented for categorization and detection. Senthilkumar et al.^21^ accumulated the Recurrent lncRNA gene expression data, missing data imputed employing the Mode and Mean Missing method (MMM-DI), and the Hilbert–Schmidt independence criterion with Diversity Artificial Fish Swarm (HSDAFS) model is utilized for the feature selection process. The ENSemble Classification Framework (ENSCF) model is employed for recurrence prediction. Seyala and Abdullah^22^ utilized cluster evaluation, employing nonparametric cubic B-spline and penalization methods such as concave penalization function, cubic spline penalty (CSP), and nonparametric pairwise grouping (NPG) techniques. Also, Bayesi, an Information Criteria (BIC) and alternative direction methods of the multiplier (ADMM) models are utilized. In^23^, the authors employed novel methodologies for transfer learning by using uncategorized medical images of the same ailments to mitigate the ImageNet impact. Mukhlif, Al-Khateeb, and Mohammed^24^ present a Dual Transfer Learning (DTL) model. The model also integrated data augmentation for class balance and sample augmentation. Pacal and Kılıcarslan^25^ implement advanced DL models, including CNN and vision transformer (ViT) methods, together with data augmentation and ensemble learning methods. In^26^, the Multi-Axis Vision Transformer (MaxViT) model is presented and optimized for Pap smear data, integrating ConvNeXtv2 and GRN-based MLPs models. Karaman et al.^27^ introduced a DL model, combining YOLOv5 object detection with artificial bee colony algorithm (ABC) optimization for polyp detection. In^28^, an ABC into the YOLO model to optimize the hyper-parameters of YOLO-based methods is presented.

## The proposed method

**Figure 1:**
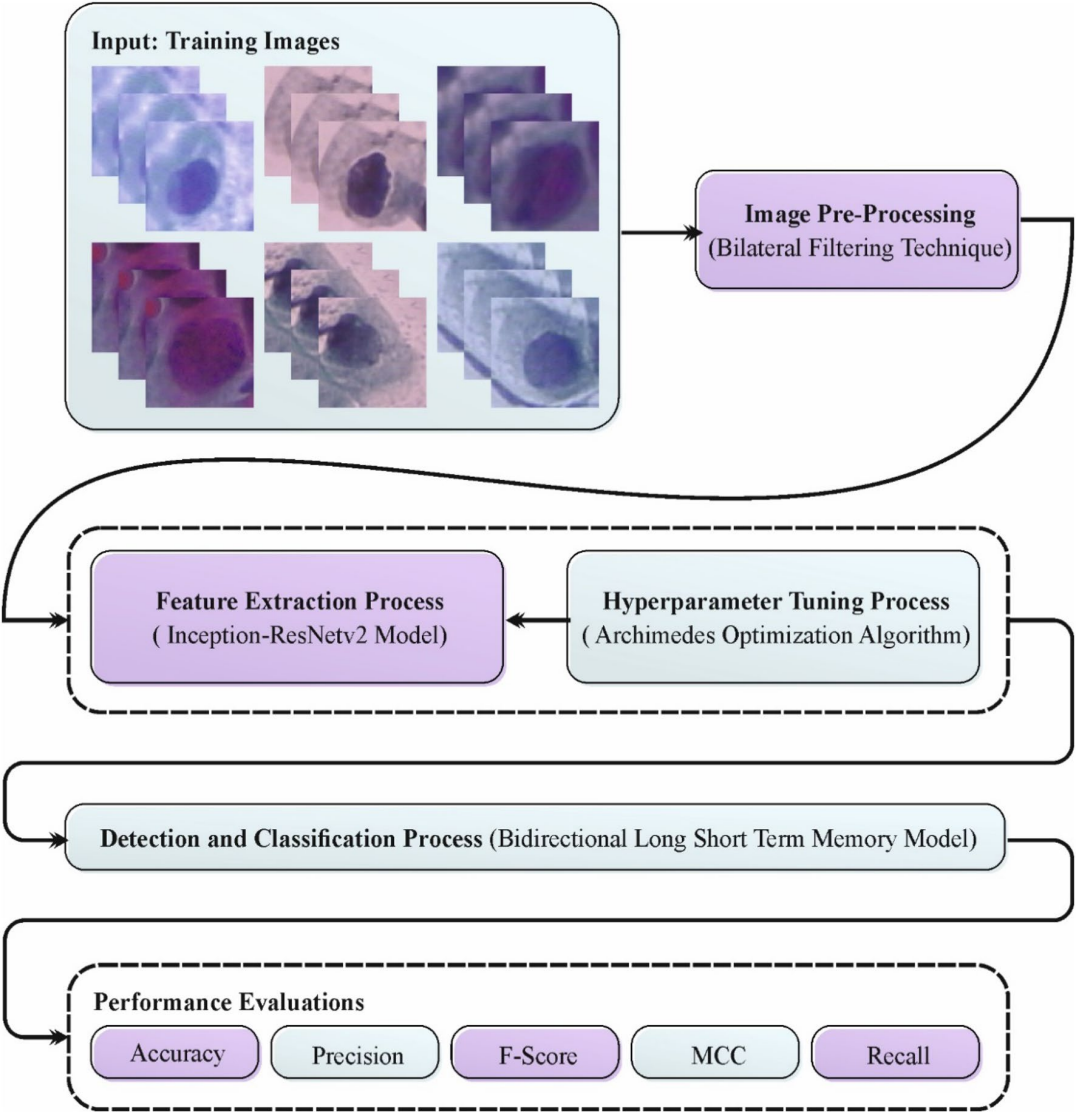
Overall flow of the CPLDC-AOATL technique.

In this study, we offer the design of an enhanced CPLDC-AOATL methodology. The system's drive is to diagnose CC on medical images. It contains four different procedures: BF-based image preprocessing, Inception-ResNetv2-based feature extractor, AOA-based hyperparameter tuning, and BiLSTM-based classification. Figure 1 illustrates the entire flow of the CPLDC-AOATL approach.

### Image preprocessing

At the preliminary stage, the CPLDC-AOATL technique involves the BF technique to eliminate the noise in the input images. BF is a low‐pass filter that smoothens an image by keeping the quality of the object's edge^29^. The reliability and effectiveness of filter in decreasing speckle:

1 $$h\left(q\right)={\Gamma }^{-1}\left(q\right){\int }{a}_{\phi \left(q\right)}f\left({\varepsilon }^{\prime}\right)c\left({\varepsilon }^{\prime},q\right)s\left(f\left({\varepsilon }^{\prime}\right),f\left(q\right)\right)d{\varepsilon }^{\prime}$$

2 $$\Gamma \left(q\right)={\int }{a}_{\phi \left(q\right)}c\left({\varepsilon }^{\prime},q\right)s\left(f\left({\varepsilon }^{\prime}\right),f\left(q\right)\right)d{\varepsilon }^{\prime}$$

where $f(q)$ implies the novel image$,$ $h(q)$ denotes the filtered image, $Q(q)$ indicates the measure of the neighbourhood window, and ${\varepsilon }^{\prime}$ refers to the pixel location. $({\varepsilon }^{\prime}, q)$ and $({\varepsilon }^{\prime}),f(q)$, correspondingly, determined as

3 $$c\left({\varepsilon }^{\prime}, {q}^{\prime}\right)=\left(\frac{-|q-{\varepsilon }^{\prime}{\| }^{2}}{2{\sigma }_{C}^{2}}\right)$$

4 $$s\left(f\left({\varepsilon }^{\prime}\right),f\left({q}^{\prime}\right)\right)=\text{exp}\left(\frac{(f(q)-f(\varepsilon ){)}^{2}}{2{\sigma }_{s}^{2}}\right)$$

where $\sigma s$ and $\sigma c$ are the standard deviation (SD) of the Gaussian random and the $\varphi$ window area.

### Inception-ResNetv2 model

**Figure 2:**
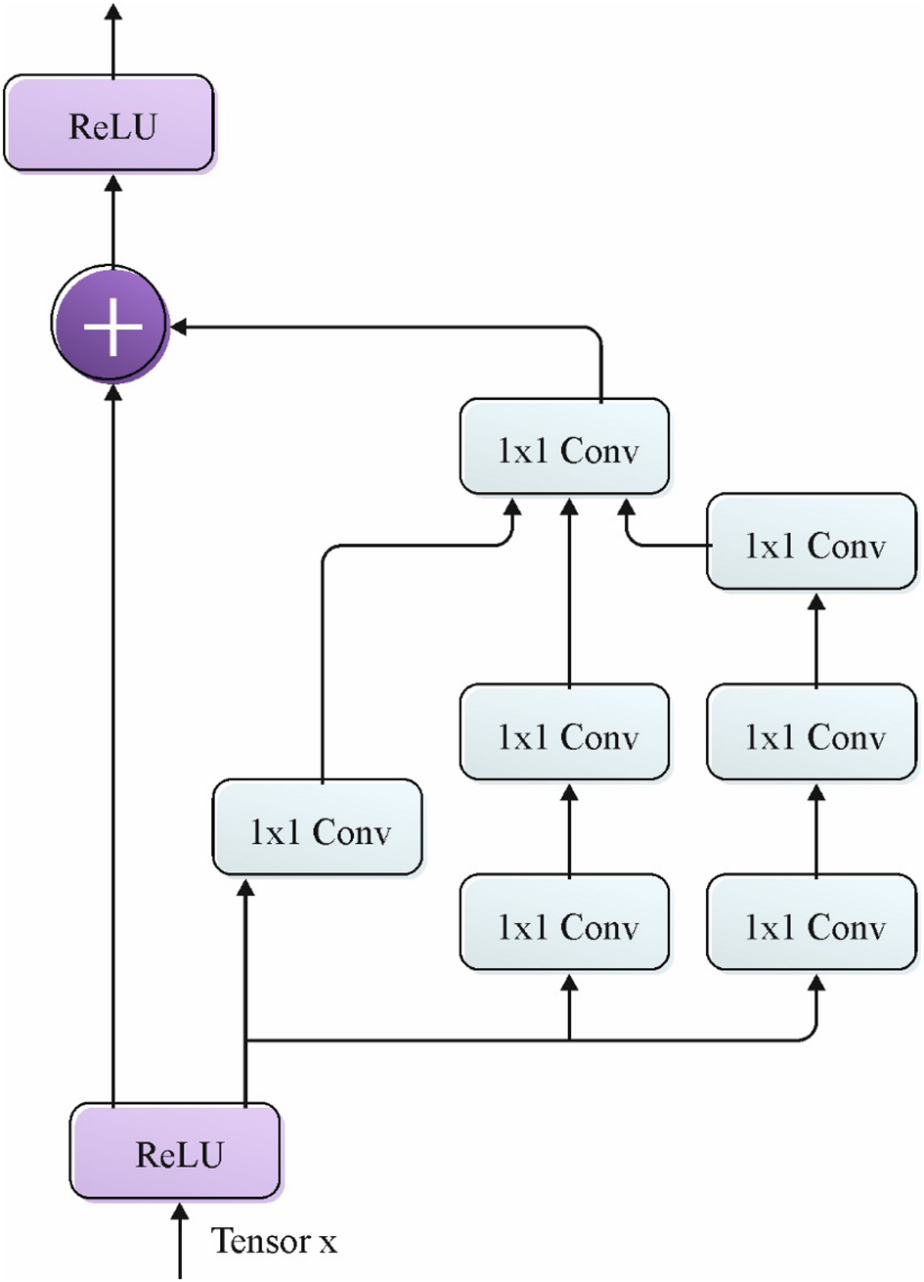
Structure of inception-ResNetV2 Model.

For the feature extraction process, the CPLDC-AOATL technique applies the Inception-ResNetv2 model. Inception‐ResNetV2 incorporates the inception model and residual network^30^. The multi-branch structures are one of the reasons for the popularity of the Inception module. In each branch, a group of filters (1 × 1, 3 × 3, 5 × 5, etc.) are integrated utilizing concatenation. The residual module can be prominent for its capability to train deep architecture. The proposed model makes effective use of residual connections. Firstly, over a million images from the ImageNet gathered are utilized for training the Inception‐ResNetV2 method. This 824‐layer network categorizes data into 1000 classes. Figure 2 demonstrates the infrastructure of the Inception‐ResNetV2 model.

Consequently, Inception-ResNetV2 has captured dynamic features representing an extensive range of images. This model requires an input image size of $299\text{x}299\text{x}3$. The dropout rate is set as 0.8. This model is finetuned based on the FC layer. The FC layer comprises 1000 object classes; the output class is four for all datasets. Thus, a new FC layer is built and detached from the preceding FC layer, which is later interconnected to the early one. The trained model is used to extract deep features. The activation can be applied, and 1536 deep features are extracted for all the images. The extracting feature was analyzed, and a few redundant data were found.

### Hyperparameter tuning using AOA

At this stage, the hyperparameters are chosen using AOA. AOA is an optimization method derived from the Archimedes’ Principle. An object is absorbed in a liquid and pushed upward by a buoyancy force^31^. This force will be equal to the mass of the dislocated liquid. As reported by this method, each object dipped in $y{n}_{a}$ mican attempts in the equilibrium condition. At this condition, the buoyant force and weight of the object become equal. It will be specified in Eq. (5).

5 $${F}_{b}={w}_{o};{p}_{b}{v}_{b}{a}_{b}={p}_{0}{v}_{o}{a}_{o}$$

Here, $0$ denotes the immersed object, $v$ represents the volume, $p$ denotes the density, $b$ specifies fluid, and $a$ represents the acceleration. In AOA, immersed objects produce a population. Primary search will be executed with random values that can be standard in most optimizer methods. Each iterative volume and density value has been upgraded until the method's end conditions are satisfied. Steps are applied in AOA as given below:

Step 1: Values of the objects have been arbitrarily allocated as in Eq. (6).
  6 $${O}_{i}=l{b}_{i}\times rand\times \left(u{b}_{i}-l{b}_{i}\right) i=\text{1,2},\dots ,N$$
  Now, ${O}_{i}$ refers to the ith object in $N$, $N$ refers to population, $u{b}_{i}$ describes the upper boundary, and $l{b}_{i}$ refers to the lower boundary. Density (*den*) and volume $(vol)$ values have been arbitrarily initialized as in Eq. (7). Acceleration $\left(ac{c}_{i}\right)$ will be represented in Eq. (8).
  7 $$de{n}_{i}=rand;vo{l}_{i}=rand$$
  8 $$ac{c}_{i}=l{b}_{i}+rand\times \left(u{b}_{i}-l{b}_{i}\right)$$
Step 2: Density and volume can be upgraded by Eq. (9).
  9 $$de{n}_{i}^{t+1}=de{n}_{i}^{t}+rand\times \left(de{n}_{best}-de{n}_{i}^{t}\right);vo{l}_{i}^{t+1}=vo{l}_{i}^{t+1}+rand\times \left(vo{l}_{best}-vo{l}_{i}^{t}\right)$$
  On the other hand, $de{n}_{best}$ and $vo{l}_{best}$ describe the best density and volume values.
Step 3: The transfer operator (TF) has been improved. Alternatively, the density factor will be reduced. This allows the exchange among steps (exploration‐exploitation) with equilibrium conditions next to the collisions. It can be achieved by the Eq. (10).
  10 $$TF=\text{exp}\left(\frac{t-{t}_{\text{max}}}{{t}_{\text{max}}}\right)$$
  Now, ${r}_{\text{max}}$ denotes the maximal number of iterations. $t$ specifies the iteration number. Density reducing factor (d) reduces over time by applying Eq. (11):
  11 $${d}^{t+1}=\text{exp}\left(\frac{{t}_{\text{max}}-r}{{t}_{\text{max}}}\right)-\left(\frac{t}{{r}_{\text{max}}}\right)$$
Step 4: Exploration stage: During the fourth step, collisions arise based on the TF value. Equation (12) will upgrade the object's acceleration $(ac{c}_{i})$.
  12 $$ac{c}_{i}^{t+1}=\frac{de{n}_{mr}+vo{l}_{mr}\times ac{c}_{mr}}{de{n}_{i}^{t+1}\times vo{l}_{i}^{t+1}}$$
  $Vo{l}_{i}$ refers to volume, and $de{n}_{i}$ means density. $ac{c}_{i}$ denotes acceleration of object $i,$ and $mr$ pointed out values of random objects.
Step 5: Exploitation stage: Based on the TF values, collisions do not occur. At this condition, Eq. (13) will upgrade the object's acceleration.
  13 $$ac{c}_{i}^{t+1}=\frac{de{n}_{best}+vo{l}_{best}\times ac{c}_{best}}{de{n}_{i}^{t+1}\times vo{l}_{i}^{t+1}}$$
  Now, $ac{c}_{best}$ describes the best acceleration values.
Step 6: Normalize acceleration step: During the 6th stage, acceleration can be extreme in settings, but the performance is distant in the global minimal and reduces over time in alternative conditions. Hence, $ac{c}_{i,norm}^{t+1}$ is adjusted to the step size variation for every object. Equation (14) will be applied.
  14 $$ac{c}_{i-norm}^{t+1}=u\times \frac{ac{c}_{i}^{t+1}-\text{min}\left(acc\right)}{\text{max}(acc)-\text{min}\left(acc\right)}+l$$
  Here, *l* and *u* define the lower and upper values.
Step 7: Update step. The locations can be upgraded. Once TF is lesser than or equal to 0.5 (exploration stage) and represented in Eq. (15)
  15 $${x}_{i}^{t+1}={x}_{i}^{t}+{C}_{1}\times rand\times ac{c}_{i-norm}^{t+1}\times d\times \left({x}_{rand}-{x}_{i}^{t}\right)$$
  whereas ${C}_{1}$ will be equal to 2. When $TF$ is higher than 0.5, the exploration stage will be performed. Object locations have been upgraded by applying Eq. (16).
  16 $${x}_{i}^{t+1}={x}_{best}^{t}+F\times {C}_{2}\times rand\times ac{c}_{i-norm}^{t+1}\times d\times \left(T\times {x}_{best}-{x}_{i}^{t}\right)$$
  Here, ${C}_{2}=6.$ $T={C}_{3}\times TF$. The value of $T$ improves with time at range $[{C}_{3}\text{x0.3,1}].$ $F$ specifies the flag parameter employed to alter the direction Eq. (17):
  17 $$F=\left\{\begin{array}{l}+1 if P\le 0.5\\ -1 if P>0.5\end{array}\right.$$
  We know that $P=2\times rand-{C}_{4}.$
Step 8: Evaluation step. The fitness function (FF) has been calculated. Once a higher result has been determined, it will be remembered.
  The AOA develops an FF to realize more excellent classifier solutions. It expresses a positive integer to imply the optimum efficiency of the candidate results. During this case, the decreasing of the classifier error values can be assumed to be FF, as defined in Eq. (18).
  18 $$fitness\left({x}_{i}\right)=ClassifierErrorRate\left({x}_{i}\right)=\frac{misclassified \; instance \;counts }{Total\; instance \;counts }\times 100$$

### Cancer detection using BiLSTM

Finally, the CPLDC-AOATL technique involves the BiLSTM model for the cancer detection process. The Bi‐LSTM depends on a DL model mainly intended to evaluate the network on numerous multipaths^32^. This model plays a vital part in each component in an input signal, which unites the related facts from both the past and present. In this situation, it makes numerous sufficient outputs. The linear DL model $fd(si, vx)={\sum }_{bc=1}^{hd}s{i}_{\text{bc}}v{x}_{bc}$, whereas the input is called $hd$, the terms fd and $vx$ signify the output and weights of the system. The Bi‐LSTM model has dual layers of LSTM on side‐to‐side arrays. The one layer in LSTM was trained beside the input series in the forward direction. The input series was assumed to be in the inverse order for training the extra LSTM layer in a backward direction direction. The LSTM system was measured to correct the gradient issue in RNNs about the more extended sequence data. It has four gates that are set in Eqs. (19), (20), (21) and (22).

19 $$r{v}_{mn}=\phi \left({R}_{rv}d{a}_{mn}+{V}_{rv}g{d}_{mn-1}+h{a}_{rv}\right)$$

20 $$v{r}_{mn}=tanh\left({R}_{vr}d{a}_{mn}+{V}_{vr}g{d}_{mn-1}+h{a}_{vr}\right)$$

21 $$t{i}_{mn}=\nu \left({R}_{ti}d{a}_{mn}+{V}_{ti}g{d}_{mn-1}+h{a}_{ti}\right)$$

22 $$h{o}_{mn}=\nu \left({R}_{ho}d{a}_{mn}+{V}_{ho}g{d}_{mn-1}+haho\right)$$

Here, ${R}_{vr},$ ${R}_{rv},$ ${R}_{ti},$ ${R}_{ho}$ denotes the weight matrices on the input condition $d{a}_{mn}$. Likewise, the weighted metrics from the preceding short-term $g{d}_{mn-1}$ are assumed as ${V}_{vr},{V}_{rv},{V}_{ti},{ and V}_{ho}.$ Here, the variables $h{a}_{vr},h{a}_{rv},h{a}_{ri},ha{h}_{0}$ are specified as bias. The present long‐term condition from the network $c{d}_{mn}$ is resultant as in Eq. (23)

23 $$c{d}_{mn}=r{v}_{mn}\otimes c{d}_{mn-1}+t{i}_{mn}\otimes v{r}_{mn}$$

Lastly, the output ${f}_{mn}$ is resultant in Eq. (24)

24 $$y{f}_{mn}=g{d}_{mn}=h{o}_{mn}\otimes tanh\left(cd\right)$$

The variable $c{d}_{mn-1}$ is stated as a preceding long-term condition.

## Performance validation

**Figure 3:**
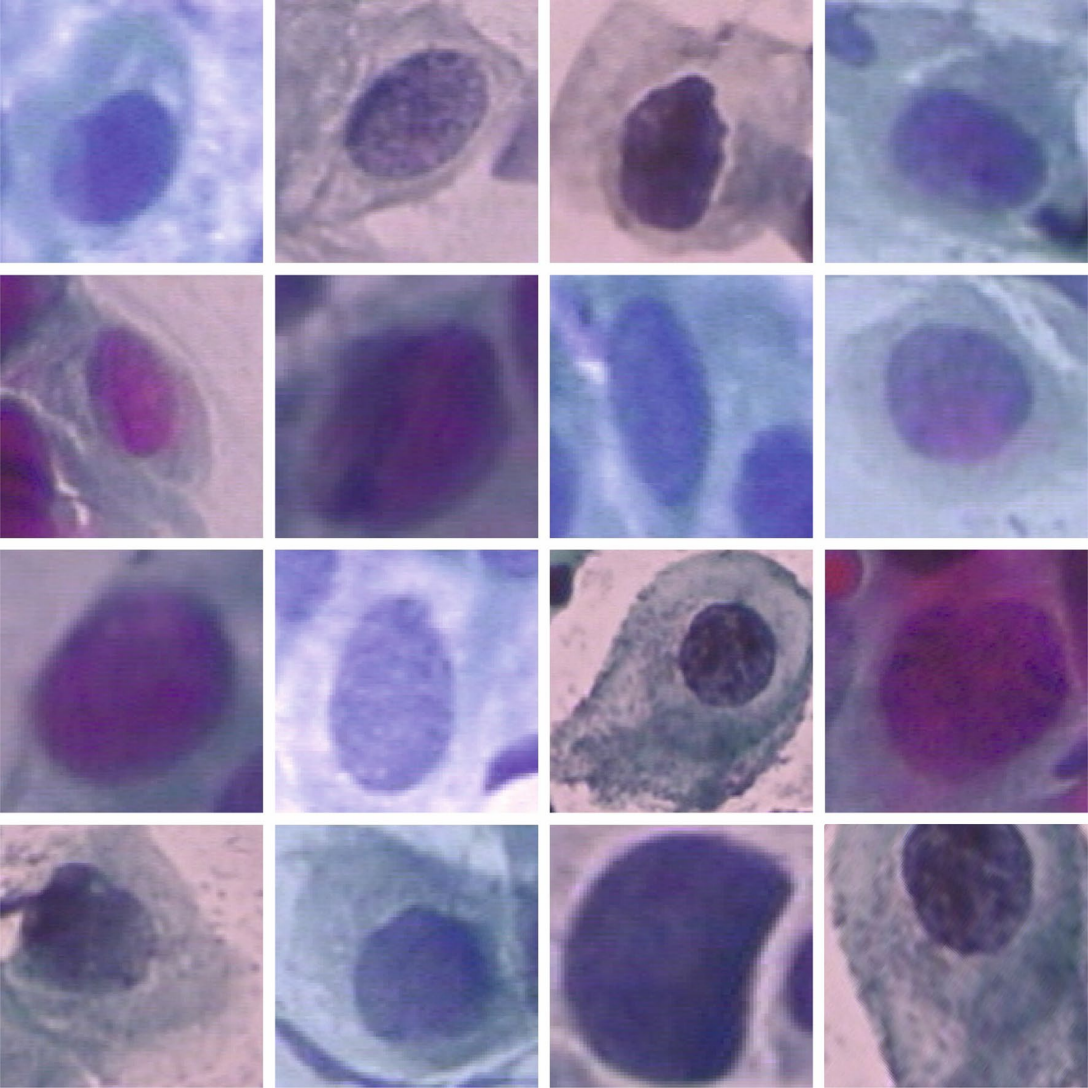
Sample images.

The performance evaluation of the CPLDC-AOATL technique is tested using benchmark dataset^33^ comprising distinct classes such as Intermediate Squamous (ISE) (70), Superficial squamous (SSE) (74), columnar (CE) (98), Moderate Dysplasia (MOS-NKD) (146), Carcinoma In Situ (SCCSI) (151), Severe Dysplasia (SS-NKD) (197), and Mild Dysplasia (MS-NKD) (182). Figure 3 illustrates the sample images.

**Figure 4:**
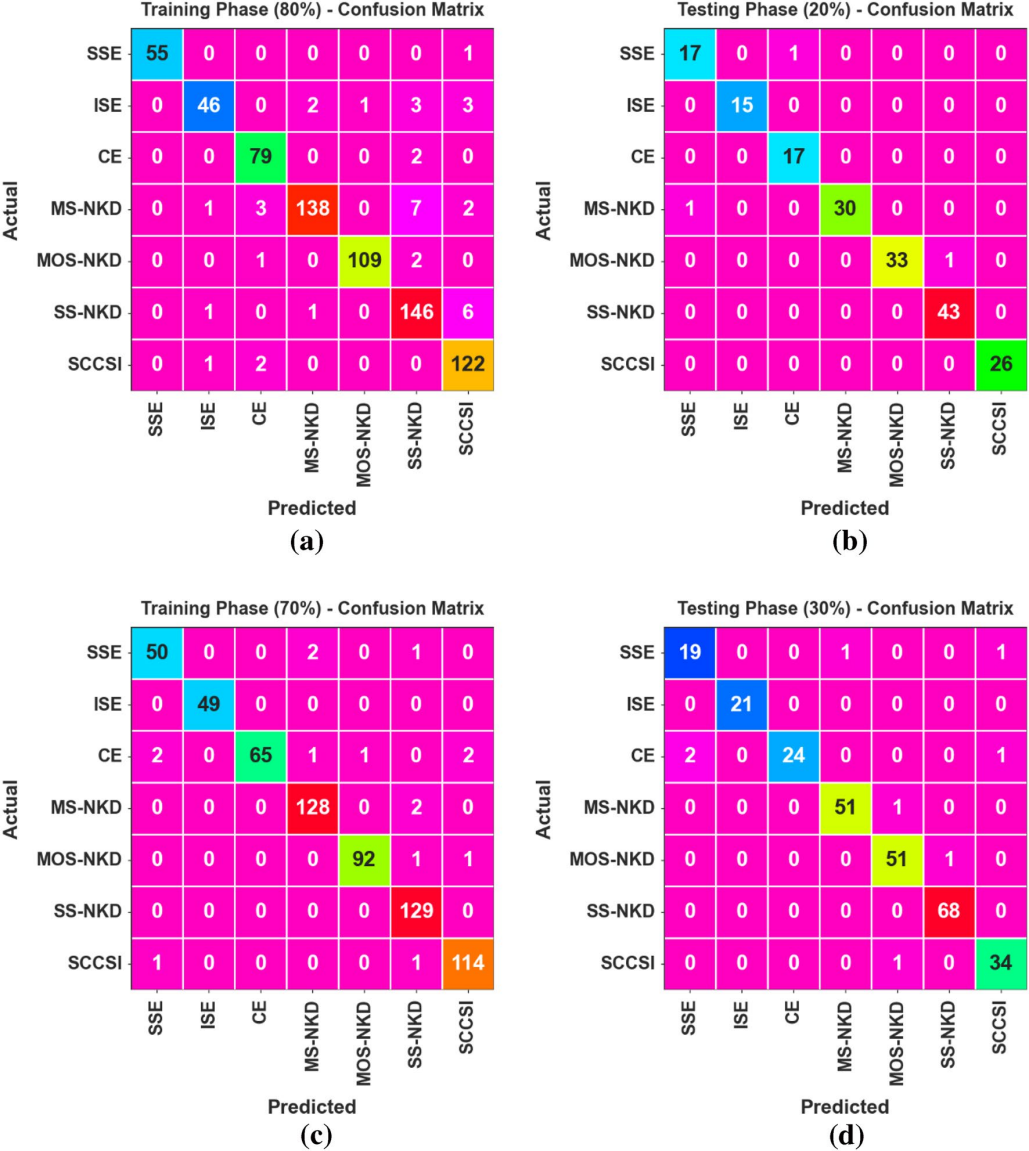
Confusion matrices of CPLDC-AOATL method (**a**,**b**) 80%TRAPH/20%TESPH and 70%TRAPH/30%TESPH.

Figure 4 displays the confusion matrices produced by the CPLDC-AOATL method on 80:20 and 70:30TRAPH/TESPH. These indicate that the CPLDC-AOATL method effectively recognizes classes.

**Figure 5:**
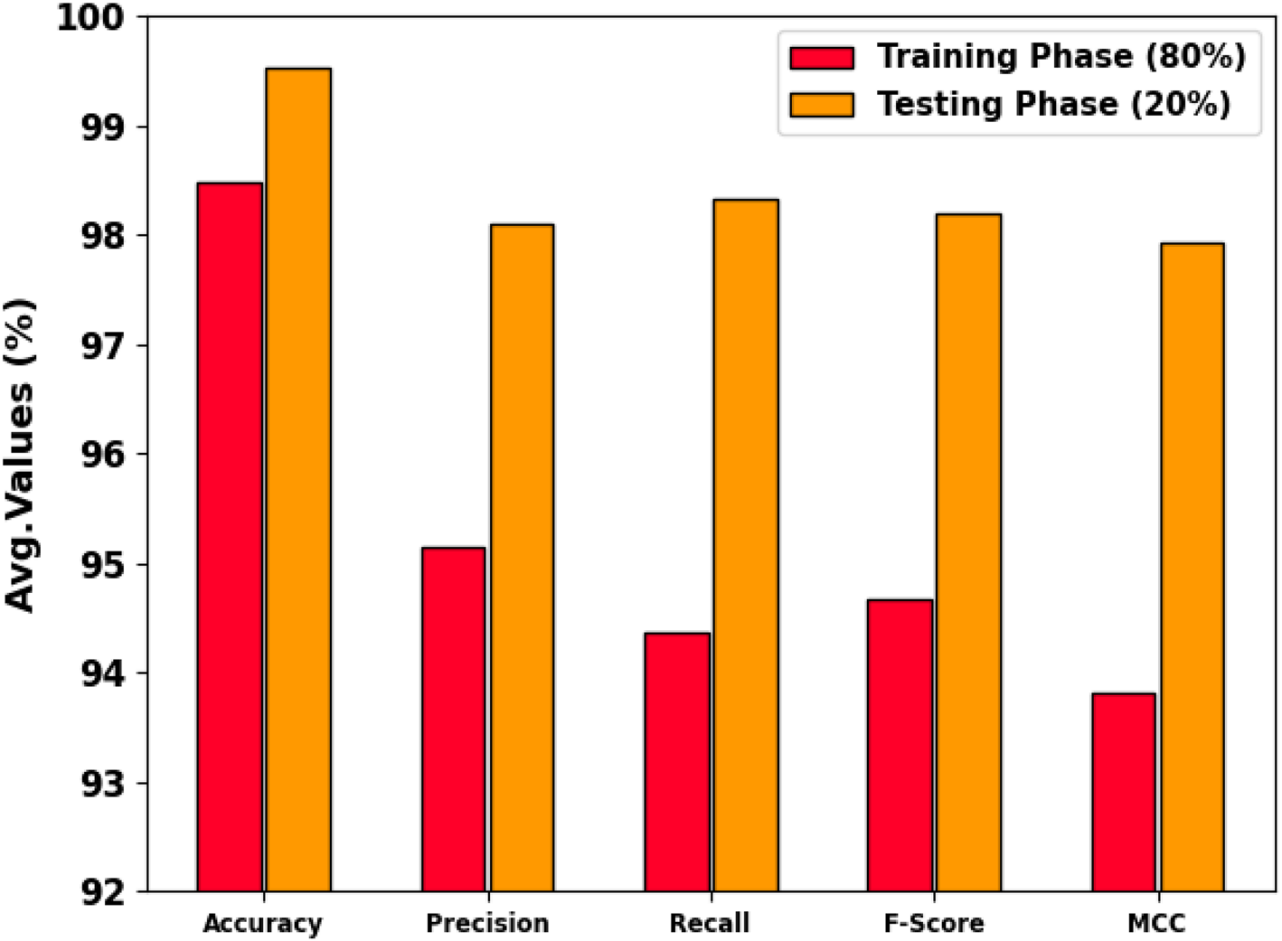
Average of the CPLDC-AOATL system on 80%TRAPH/20%TESPH.

The cancer recognition output of the CPLDC-AOATL technique at 80%TRAPH and 20%TESPH is made in Table 1 and Fig. 5. The obtained values state that the CPLDC-AOATL technique reaches effectual performance. With 80%TRAPH, the CPLDC-AOATL technique gains an average $acc{u}_{y}$ of 98.48%, $pre{c}_{n}$ of 95.15%, $rec{a}_{l}$ of 94.36%, ${F}_{score}$ of 94.67%, and MCC of 93.81%. Also, based on 20%TESPH, the CPLDC-AOATL method acquires an average $acc{u}_{y}$ of 99.53%, $pre{c}_{n}$ of 98.09%, $rec{a}_{l}$ of 98.33%, ${F}_{score}$ of 98.19%, and MCC of 97.93%, respectively.

**Table 1:** Cancer recognition outcome of CPLDC-AOATL model under 80%TRAPH/20%TESPH.

Class labels	$Acc{u}_{y}$	$Pre{c}_{n}$	$Rec{a}_{l}$	${F}_{score}$	*MCC*
TRAPH (80%)					
SSE	99.86	100	98.21	99.10	99.03
ISE	98.37	93.88	83.64	88.46	87.75
CE	98.91	92.94	97.53	95.18	94.60
MS-NKD	97.82	97.87	91.39	94.52	93.25
MOS-NKD	99.46	99.09	97.32	98.20	97.88
SS-NKD	97.00	91.25	94.81	92.99	91.12
SCCSI	97.96	91.04	97.60	94.21	93.05
Average	98.48	95.15	94.36	94.67	93.81
TESPH (20%)					
SSE	98.91	94.44	94.44	94.44	93.84
ISE	100.00	100.00	100.00	100	100.00
CE	99.46	94.44	100	97.14	96.89
MS-NKD	99.46	100.00	96.77	98.36	98.05
MOS-NKD	99.46	100.00	97.06	98.51	98.19
SS-NKD	99.46	97.73	100.00	98.85	98.51
SCCSI	100.00	100.00	100.00	100.00	100.00
Average	99.53	98.09	98.33	98.19	97.93

**Figure 6:**
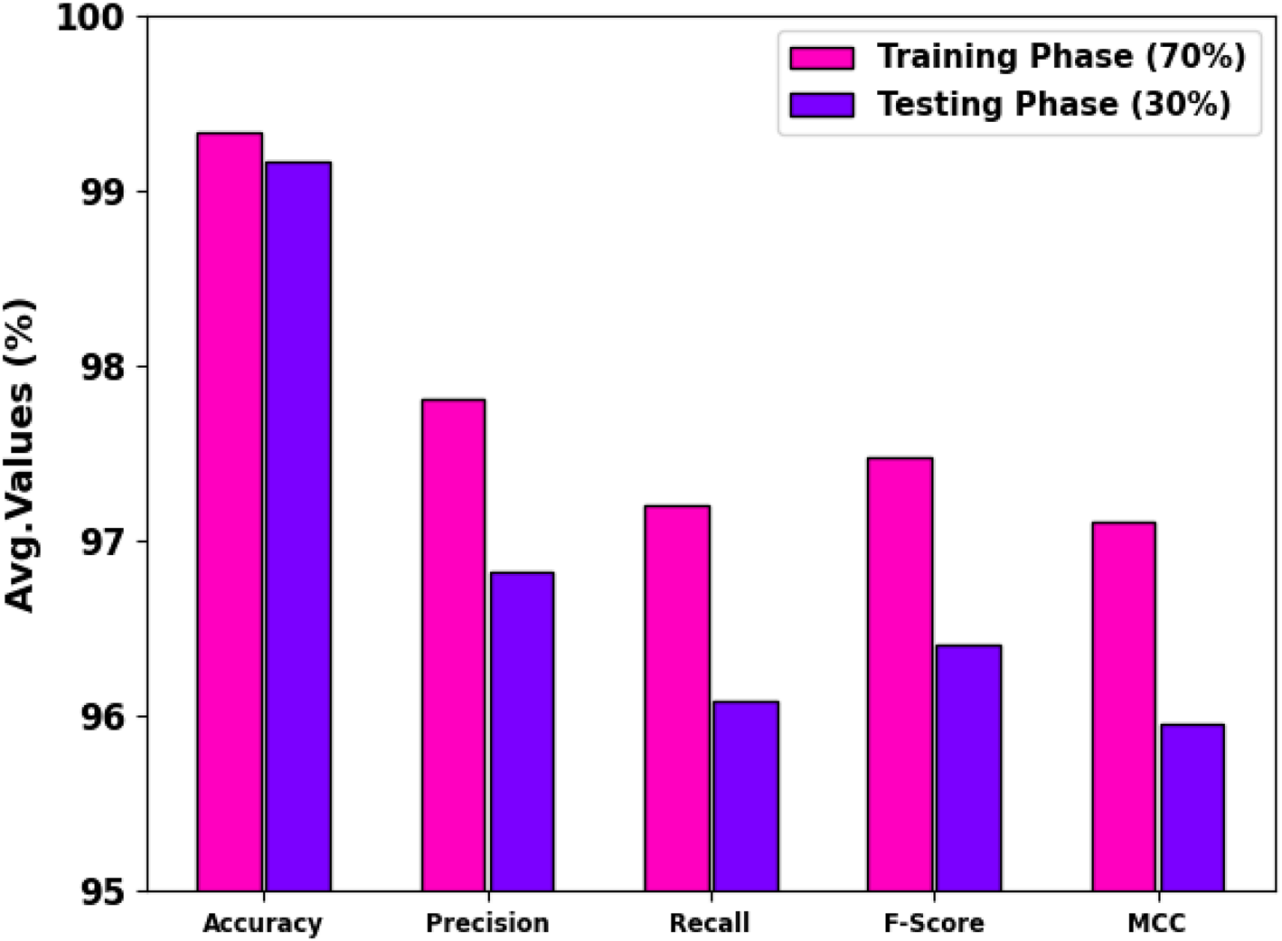
Average of the CPLDC-AOATL system on 70%TRAPH/30%TESPH.

The cancer recognition results of the CPLDC-AOATL method at 70%TRAPH and 30%TESPH are described in Table 2 and Fig. 6. These achieved values specified that the CPLDC-AOATL system acquires proficient performance. Based on 70%TRAPH, the CPLDC-AOATL algorithm provides an average $acc{u}_{y}$ of 99.33%, $pre{c}_{n}$ of 97.81%, $rec{a}_{l}$ of 97.21%, ${F}_{score}$ of 97.48%, and MCC of 97.11%. Similarly, with 30%TESPH, the CPLDC-AOATL system achieves an average $acc{u}_{y}$ of 99.17%, $pre{c}_{n}$ of 96.82%, $rec{a}_{l}$ of 96.09%, ${F}_{score}$ of 96.41%, and MCC of 95.96%, correspondingly.

**Table 2:** Cancer recognition outcome of CPLDC-AOATL method at 70%TRAPH/30%TESPH.

Class labels	$Acc{u}_{y}$	$Pre{c}_{n}$	$Rec{a}_{l}$	${F}_{score}$	*MCC*
TRAPH (70%)					
SSE	99.07	94.34	94.34	94.34	93.83
ISE	100.00	100.00	100.00	100.00	100.00
CE	99.07	100.00	91.55	95.59	95.18
MS-NKD	99.22	97.71	98.46	98.08	97.60
MOS-NKD	99.53	98.92	97.87	98.40	98.12
SS-NKD	99.22	96.27	100.00	98.10	97.64
SCCSI	99.22	97.44	98.28	97.85	97.38
Average	99.33	97.81	97.21	97.48	97.11
TESPH (30%)					
SSE	98.55	90.48	90.48	90.48	89.69
ISE	100.00	100.00	100.00	100.00	100.00
CE	98.91	100.00	88.89	94.12	93.72
MS-NKD	99.28	98.08	98.08	98.08	97.63
MOS-NKD	98.91	96.23	98.08	97.14	96.48
SS-NKD	99.64	98.55	100.00	99.27	99.03
SCCSI	98.91	94.44	97.14	95.77	95.16
Average	99.17	96.82	96.09	96.41	95.96

**Figure 7:**
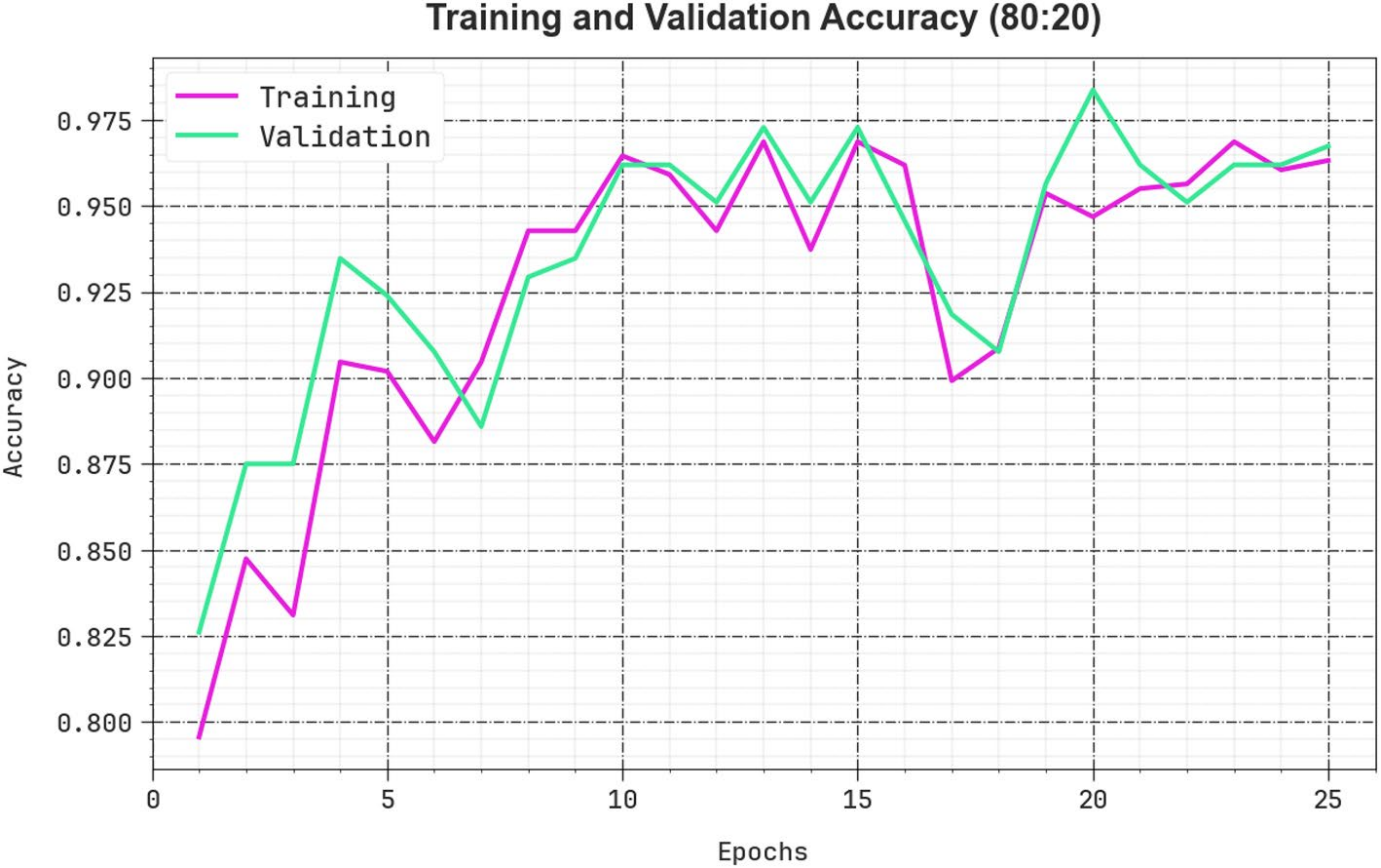
$Acc{u}_{y}$ curve of the CPLDC-AOATL model at 80%TRAPH/20%TESPH.

The efficiency of the CPLDC-AOATL method on 80%TRAPH/20%TESPH is demonstrated in Fig. 7 in the form of training accuracy (TRAA) and validation accuracy (VALA) curves. This figure displays a valuable analysis of the behaviour of the CPLDC-AOATL algorithm over varying epoch counts, demonstrating its learning process and generalization capabilities. The figure shows a continuous enhancement in the TRAA and VALA, with progress in epochs. It ensures the adaptive aspects of the CPLDC-AOATL technique in the pattern recognition process with TRA and TES data. The improved trends in VALA summarize the capability of the CPLDC-AOATL system to adjust to the TRA data and provide correct classification of undetected data, specifying robust generalization abilities.

**Figure 8:**
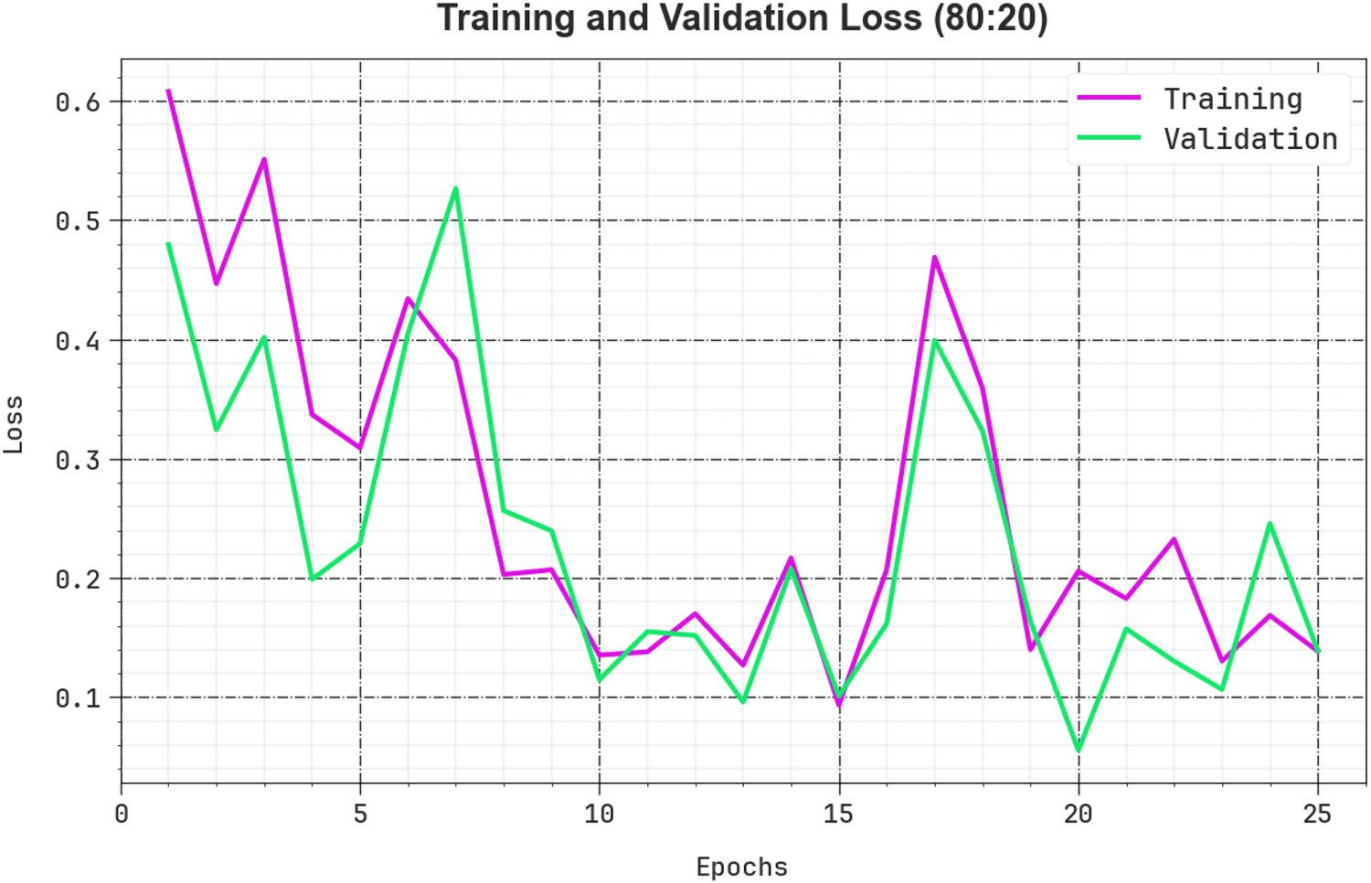
Loss curve of the CPLDC-AOATL method at 80%TRAPH/20%TESPH.

Figure 8 illustrates a wide-ranging representation of the training loss (TRLA) and validation loss (VALL) results of the CPLDC-AOATL technique with 80%TRAPH/20%TESPH over distinct epochs. The progressive decreases in TRLA highlight the CPLDC-AOATL technique, increasing the weights and diminishing the classification error under TRA and TES data. The figure specifies a clear understanding of the CPLDC-AOATL system related to the TRA data, highlighting its proficiency in capturing patterns. Significantly, the CPLDC-AOATL model continually raises its parameters to lessen the variances among the prediction and real TRA class labels.

**Figure 9:**
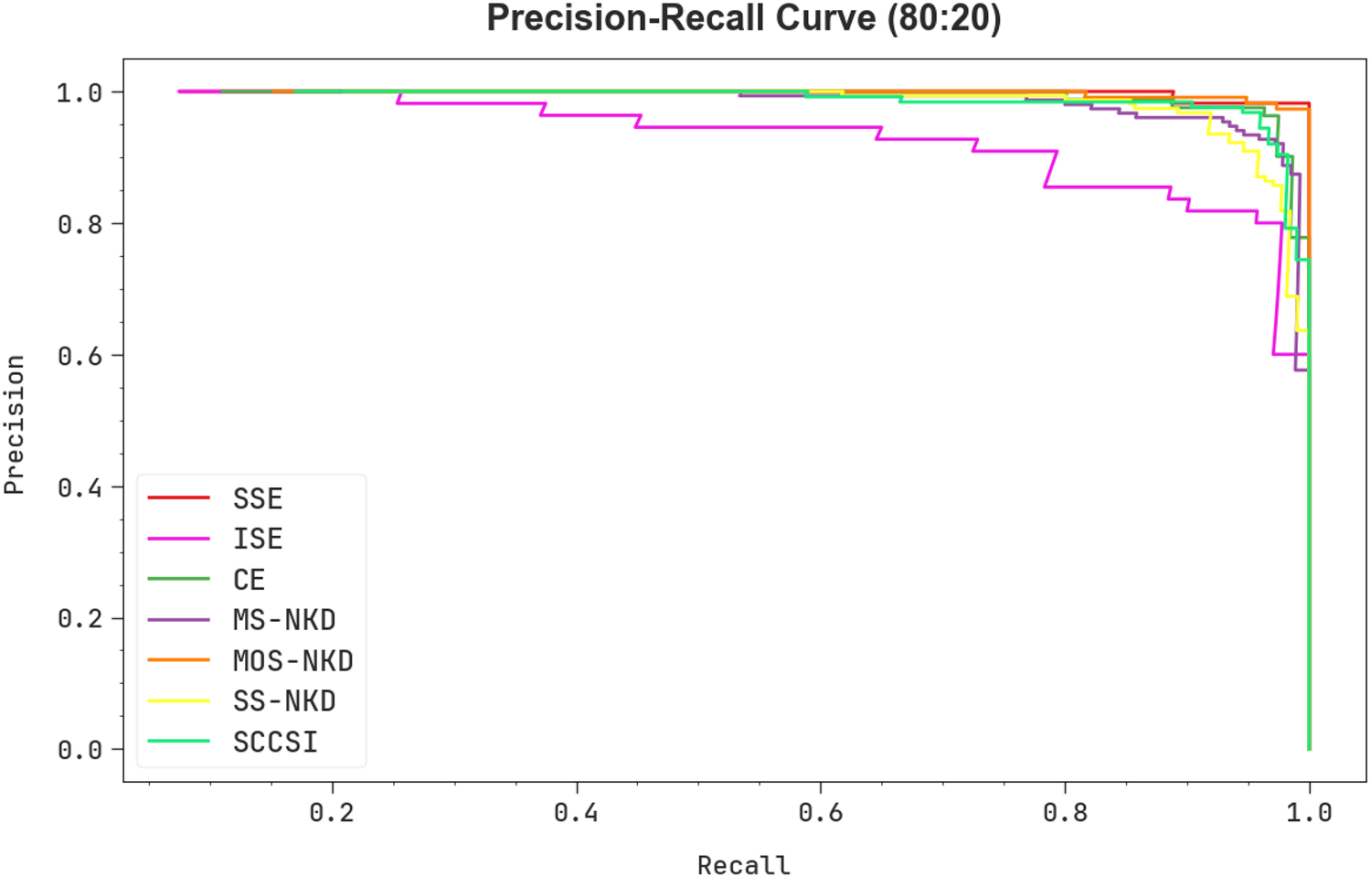
PR curve of the CPLDC-AOATL technique under 80%TRAPH/20%TESPH.

Examining the PR curve, as reported in Fig. 9, the results ensured that the CPLDC-AOATL technique at 80%TRAPH/20%TESPH gradually achieves boosted PR values in every class. It verifies the improved abilities of the CPLDC-AOATL algorithm in identifying distinct classes, demonstrating proficiency in the recognition of classes.

**Figure 10:**
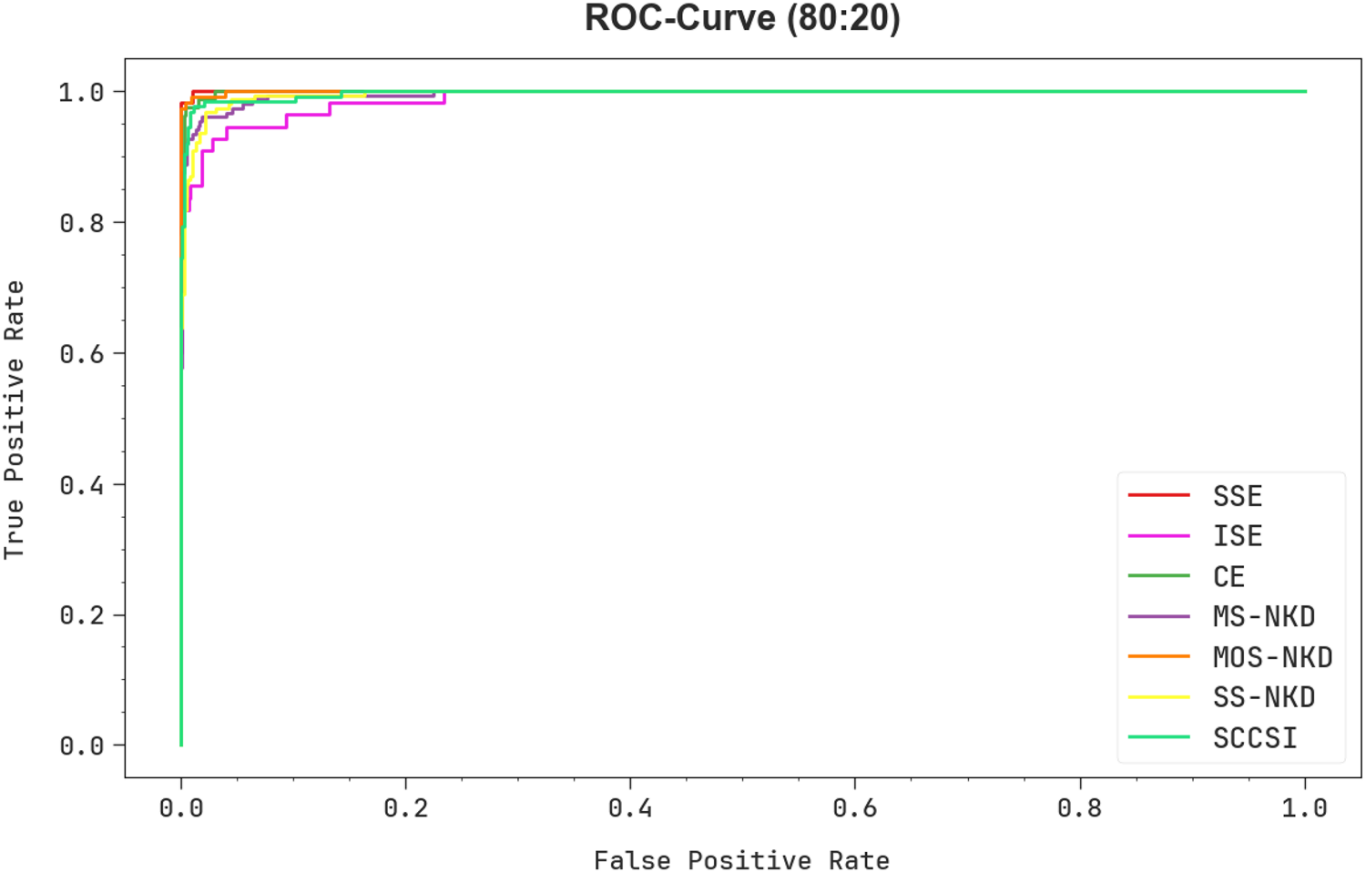
ROC curve of the CPLDC-AOATL algorithm on 80%TRAPH/20%TESPH.

Furthermore, in Fig. 10, ROC curves made by the CPLDC-AOATL method on 80%TRAPH/20%TESPH exceeded the classification of distinct labels. It gives a detailed understanding of TPR and FRP tradeoffs over distinct recognition threshold values and epoch counts. The figure pointed out the increased classifier results of the CPLDC-AOATL method with all classes, describing the efficiency of addressing diverse classification complexities.

**Figure 11:**
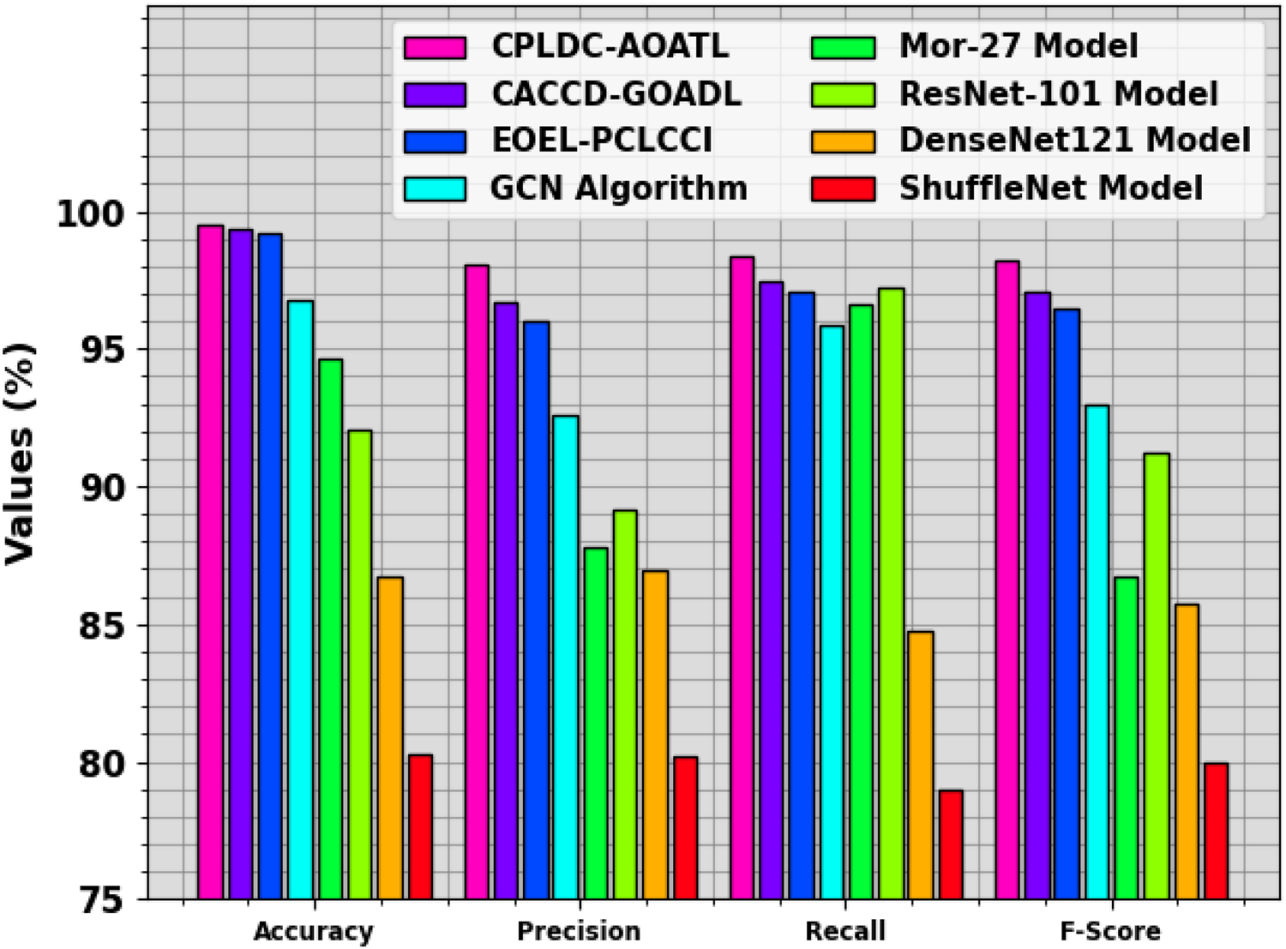
Comparative outcomes of the CPLDC-AOATL method with recent existing models.

The comparative outcomes of the CPLDC-AOATL technique are reported in Table 3 and Fig. 11^11^. These experiment outcome values indicate that the ShuffleNet and DenseNet systems have the lowest performance. Along with that, the Mor-27 and ResNet-101 techniques have reported slightly boosted results. Moreover, the CACCD-GOADL, EOEL-PCLCCI, and GCN methods have obtained closer performance. Nevertheless, the CPLDC-AOATL technique demonstrates superior performance with a maximum $acc{u}_{y}$ of 99.53%, $pre{c}_{n}$ of 98.09%, $rec{a}_{l}$ of 98.33%, and ${F}_{score}$ of 98.19%. Therefore, the CPLDC-AOATL technique can enhance the CC detection process.

**Table 3:** Comparative outcomes of the CPLDC-AOATL model with recent existing methods.

Methods	$Acc{u}_{y}$	$Pre{c}_{n}$	$Rec{a}_{l}$	${F}_{score}$
CPLDC-AOATL	99.53	98.09	98.33	98.19
CACCD-GOADL	99.39	96.73	97.46	97.06
EOEL-PCLCCI	99.18	96.04	97.06	96.47
GCN	96.75	92.59	95.86	92.95
Mor-27	94.61	87.77	96.59	86.70
ResNet101	92.02	89.20	97.23	91.22
DenseNet121	86.73	86.97	84.73	85.75
ShuffleNet	80.27	80.20	78.97	79.99

## Conclusion

This study presents the design of enhanced CPLDC-AOATL methodology. The CPLDC-AOATL approach aims to diagnose CC on the medical images. It contains four different procedures: BF-based image preprocessing, Inception-ResNetv2-based feature extractor, AOA-based hyperparameter tuning, and BiLSTM-based classification. At the preliminary stage, the CPLDC-AOATL technique involves the BF technique to eliminate the noise in the input images. Besides, the CPLDC-AOATL technique applies the Inception-ResNetv2 model for the feature extraction process, and the use of AOA chose the hyperparameters. For the cancer detection process, the CPLDC-AOATL technique involves the BiLSTM model. The experimental values of the CPLDC-AOATL method are based on a benchmark database. The experimental outcome of the CPLDC-AOATL technique emphasized the superior accuracy outcome of 99.53% over other existing approaches. The limitations of the CPLDC-AOATL method encompass its dependability on specific pre-trained techniques and datasets and future studies on integrating multi-modal data sources and improving the interpretability for facilitating clinical adoption.

## Data Availability

The data supporting this study's findings are openly available in MDE-LAB at http://mde-lab.aegean.gr/index.php/downloads, reference number^33^.
